# Organized violence as a never-ending story? Reflections in light of the Russian aggression against Ukraine

**DOI:** 10.3389/fsoc.2022.952209

**Published:** 2022-08-24

**Authors:** Ludger Pries

**Affiliations:** Faculty of Social Science, Ruhr University Bochum, Bochum, Germany

**Keywords:** organized violence, violence, modernization, not violence-intensive social spaces, violence-intensive social spaces

## Abstract

During the last decade, especially with the Russian aggression against Ukraine, armed conflicts and other forms of organized violence increased in volume and public discourse. In Sociology, violence and, particularly organized violence were marginalized topics for a long time, not least because many sociologists considered violence as a relic of traditional societies. Based on an analysis of major sociological studies and an empirical analysis of violence-related mass data, we argue that (1) violence and organized violence are not vanishing, but are genuine components of human coexistence, (2) especially in Europe, we experienced (or at least perceived) some seven decades of living in social spaces without high levels of violence, (3) other world regions are violence-intensive social spaces for generations, and (4) in light of the aggression of Russian troops against Ukraine and further challenges to come, Sociology should intensify theoretical and empirical efforts in this field of research. We first sketch out some recent trends of organized violence and related social science debates, then summarize important sociological concepts of violence and organized violence, propose to differentiate between *not violence-intensive social spaces* (NoViSS) and *violence-intensive social spaces* (ViSS) and exemplify this distinction by some global data, and finally draw some conclusions for further research on organized violence in selected fields like forced migration.

## Introduction

With the aggression of the Russian government against the sovereign state of Ukraine, the topic of armed conflicts and organized violence entered the middle of European public attention. After the systematic genocide and crimes against humanity of the German Nazi-regime during World War II, for more than 70 years, Europe had seemed to be a continent almost free of protracted larger violent conflicts. Genocide like in Rwanda in 1994, military conflicts like in Iraq from 2003 to 2011, or the declaration of an Islamic State of Iraq and the Levant in 2013 were interpreted mainly as “outdated religious” or “pre-modern” conflicts. China's violent oppression of the Uighurs or the Russian aggression against Georgia and the Crimea was considered far away or marginal. Even the Balkan wars during the 1990s were mostly categorized as ephemeral events in the transition from former socialist Yugoslavia to new independent liberal states. Topics of organized violence and longer during armed conflicts were not prominent in sociology and social sciences in Europe. In light of the Russian war against Ukraine, that many scientists and specialists of international and humanitarian law consider genocide and crimes against humanity, it is necessary to reflect on the significance of violence in sociology: How is violence, especially, organized violence approached in sociology? Why was it almost marginalized as a topic in sociology, especially in Europe? How could we develop conceptual framings for analyzing organized violence nowadays?

The main arguments to develop are: first, violence and also organized violence are not vanishing, but the genuine components of human coexistence, and to understand and analyze this, conceptual frameworks are needed. Second, in Europe, we experienced (or at least perceived) some seven decades of living in social spaces without high levels of violence. However, other world regions have to be characterized as violence-intensive social spaces for generations. Third, in light of the aggression of Russian troops against Ukraine, European sociology should intensify theoretical and empirical efforts in this field of research. In what follows, we first sketch out some recent trends of organized violence and related social science debates (Section Introduction). We then summarize important sociological concepts of violence and organized violence (Section Recent social science debates and global trends of (organized) violence). Finally, we propose to analyze organized violence empirically in a sociological perspective on everyday life in social spaces as a continuum between *non violence-intensive social spaces* (NoViSS) and *violence-intensive social spaces* (ViSS) and exemplify this distinction by some global data (Section Organized violence in social spaces of everyday life). This leads to some conclusions for further research on organized violence in selected fields like forced migration (Section Concluding remarks).^1^

## Recent social science debates and global trends of (organized) violence

When Steven Pinker published his book “The Better Angels of our Nature. Why Violence has Declined” in 2011, it was received as a provocation. While in public discourse violence and death tolls in their individual and collective forms seemed to spread all over and make the world increasingly violent, Pinker argued that during the last two thousand years the world had become more peaceful. Obviously, as Pinker himself admitted, for such a long period “we will never really know which was the worst century, because it's hard enough to pin down death tolls in the twentieth century, let alone earlier ones” (Pinker, 2011, p. 193). The research project and related website on “necrometrics” are more cautious and argue: “There are undoubtedly many other events that were never recorded and have now faded into the oblivion of forgotten history. This makes it difficult to prove whether brutality is waxing or waning in the long term. Maybe the twentieth century really *was* more barbaric than previous centuries (as some people say), but you will need more complete statistics to prove it.”^2^

**Figure 1:**
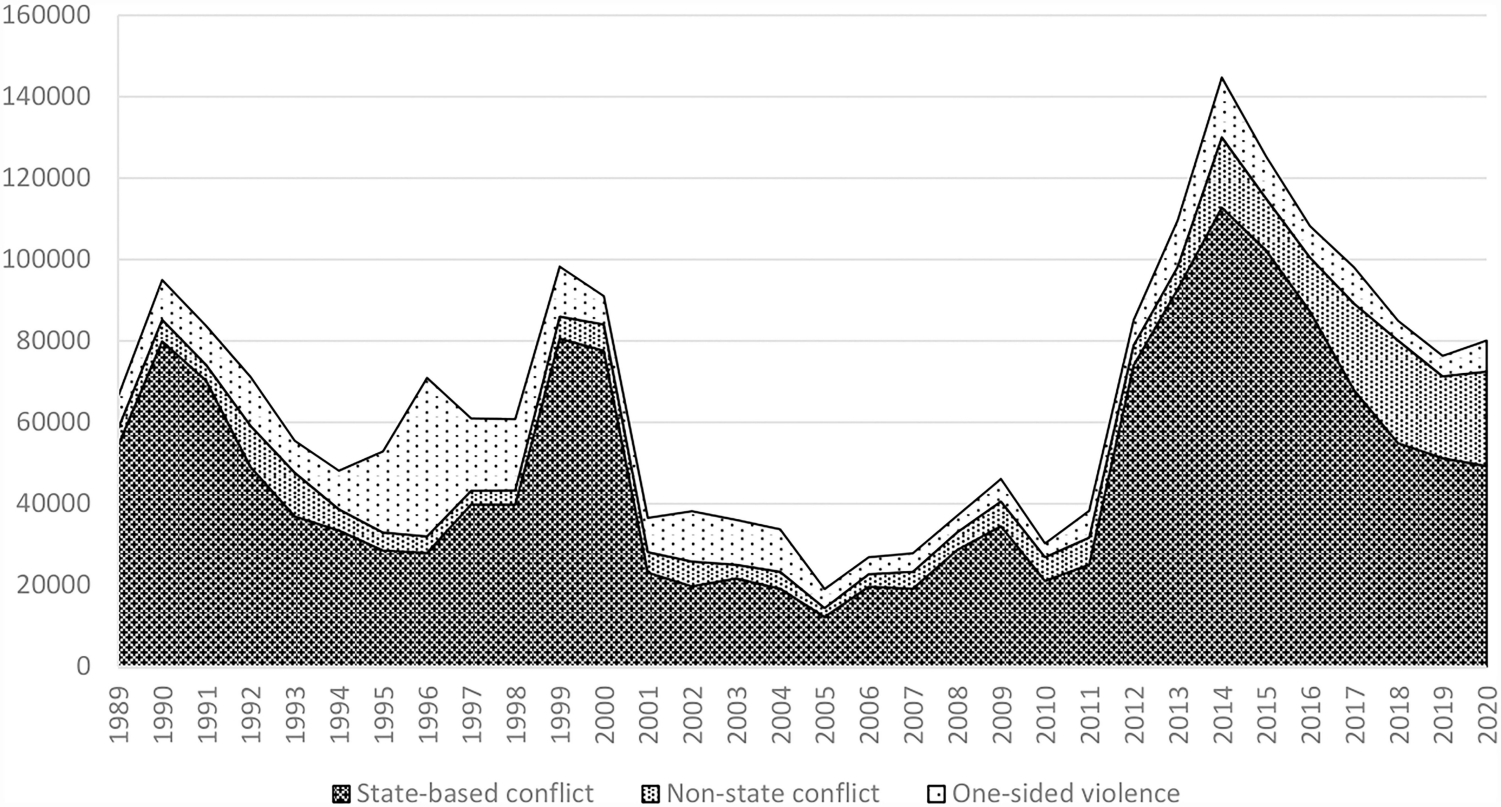
Fatalities by type (world without Rwanda in 1994, 1989–2020). Source: Own elaboration based on https://ucdp.uu.se/downloads/charts/#

There are two basic data sources that cover violence and especially organized violence in a broader spatial and time frame: data from the United Nations Office on Drugs and Crime (UNODC) and the Uppsala Conflict Data Program (UCDP). UNODC registers victims of intentional homicide in general, that is, fatalities of organized and individual violence. Although from 1990 to 2018 the world-wide intentional homicide rate decreased from some 6.8 to 5.8 homicides per 100,000 inhabitants, in several regions and countries it increased substantially, as will be shown in more detail in Section Organized violence in social spaces of everyday life.^3^ Having a closer look at the numbers of fatalities caused by state-based armed conflicts, non-state conflicts, and one-sided violence, as the definition and subtypes of *organized violence* used by the Uppsala Conflict Data Program (UCDP), there is a clear trend for the twenty-first century. Taking the UCDP data as the most trustable and considering the number of armed conflicts during the period from 1946 to 2018, the volume of conflicts more than doubled. Especially intrastate and internationalized intrastate conflicts increased. Since the beginning of the twenty-first century, the number of conflicts based on organized violence and fatalities increased substantially. This is mainly due to state-based conflicts, but the number of non-state conflicts also increasingly caused fatalities (Figure 1). In a long term view, the number of armed conflicts increased since the end of World War II consisting mainly of conflicts about territory and government.^4^

These data are coherent with theses of new types of war and organized violence that transcend the level of organized armies and troops (Kaldor, 2012; see next section for more details). But organized violence is more complex than only expressed in military and armed groups. In some regions like Central America, there are complex mixtures of corrupt or fraudulent governments, security forces, paramilitaries, death squads, youth gangs, guerrilla groups, and drug cartels. Such phenomena often go hand in hand with fragile states in the sense of lacking administrative efficiency, low levels of legitimacy, and few capabilities to control violence. The continuity of organized violent conflicts and/or of (post- or neo-)colonial external control and exploitation often correlate with such fragile states (Bourgois, 2001; Cruz, 2011; Bowen, 2019).^5^ In youth gangs, criminal networks or even centralized organizations of drug cartels, organized violence often has, although illegal, high legitimacy in general or at least in selected groups of local populations as means of collective protection against other violent collective actors or income and reputation (Cruz, 2011; Córdova, 2019; Fuerte et al., 2019). These types of organized violence, especially as non-state violence as shown in Figure 1, increased in their relative relevance for causing fatalities.

At the same time, organized violent groups and organizations also are increasingly important as subcontractors or as counterforces of legal state authorities. In the case of Ukraine, political and armed forces organized or supported by the Russian government were the backbone to declare the autonomy of Luhansk and Donezk. The Russian government already used similar strategies for invading Georgia and Crimea. Clearly, this tactic is no coincidence, but an integral part of the Russian playbook, as Russia even signaled such measures to threaten Moldova during the invasion of Ukraine.

Sociology as a discipline is essential for analyzing and understanding such complex interplay of legal and illegal, legitimate and illegitimate, formal and informal use of organized violence in multiple entanglements of economic, political, social, and cultural interests in all its complexity (for a general sociological attempt: Schetter and Müller-Koné, 2021; for Latin America: Vilalta, 2020; for Mexico: Ernst, 2015). To many, organized violence is not just an alien or exceptional event but an inextricable part of everyday life as a victim, witness, or wrongdoer. In countries like El Salvador (Bourgois, 2001), Mali and Niger (Raineri and Strazzari, 2021), Mexico (Moon and Treviño-Rangel, 2020), or Iraq (Shalash et al., 2021), (transnational) violent groups organize relevant parts of economic life and cause political struggles at least regionally.^6^ They are at the core of illegal activities like smuggling of drugs, weapons, and humans or trafficking persons (Palacios, 2015; Barnes, 2017). The different forms of organized violence should not only be counted as expressed in numbers of death fatalities but in a broader scope of consequences in and for everyday life like limiting democracy, security, accountability, and sustainable development in general. Organized violence is affecting people's everyday social practices, for El Salvador, Bourgois (2001, p. 8) uses the term everyday violence. It creates millions of traumas and other damages less visible than mortalities. Organized violence not only kills literally, but destroys the minimum conditions of a decent life, and it kills hope and health (Keller et al., 2017; König and Reimann, 2018).

Such an extended approach on organized violence is also important when looking at refuge and forced migration. For 2021 the UNHCR calculated some 97 million persons of concern, consisting of some 21 million international refugees and 49 million internally displaced persons (IDP), 4.8 million asylum seekers, 6.2 million returned asylum seekers and returned IDPs, and more than 12 million other persons of concern (UNHCR, 2021, pp. 10f). From 2010 to 2020, the growth of the world population was about 12%, international migration increased by 27%, and forced migration, counted as persons of concern defined by UNHCR, grew by 123%.^7^ “Between 2000 and 2009, the numbers of displaced generally ranged between 37 and 42 million. The last decade, however, brought a major shift. More people sought refuge, but those who had been displaced had fewer options for rebuilding their lives. As wars and conflicts dragged on, fewer refugees and internally displaced people were able to return home, countries accepted a limited number of refugees for resettlement and host countries struggled to integrate displaced populations” (UNHCR, 2020, p. 11). This dynamic leads to a downward spiral, where organized violence not only leads to forced displacement but hinders people to return, increases conflicts for resources, and cumulates conflicts in other places. Ríos (2014, p. 2006) found for Mexico: “Migration outflows are higher in places with higher drug-related violence and crime, even accounting for factors such as employment and human capital.”

As these complex entanglements are difficult to measure, the numbers of fatalities and registered conflicts as counted by UNODC and UDCP are only rough indicators of organized violence. Calculating the number of fatalities to be about 100.000 due to organized violence each year, the number of forced migrants is some 700 times higher. Additionally, IDPs and international refugees are not affected by organized violence just once in their lifetime, but they have to cope with violence in their regions of departure, on their route in transit countries, and often also in countries of arrival (Slack and Whiteford, 2011; Ríos, 2014).

All this advocates for reflecting the general role of violence in sociology. Is violence and especially organized violence increasingly fenced in and decreasing as Pinker suggested? Or will it be one of the major “pandemics” and challenges of this century? For Europe, the Russian aggression against Georgia in 2008 and Crimea in 2013 could have been perceived as wake up calls, but it was until the invasion of Ukraine in 2022 and the threatening of Moldova and other independent states that organized violence arrived in the center of public and scientific attention, and it is foreseeable that the effects of climate change will worsen hardships and violent conflicts, e.g., concerning the use of water, dams, and land. In 2022 even in Europe, organized violence is anything but an ephemeral and exceptional part of social spaces anymore. For decades, the Global North widely neglected organized violence as a crucial part of theoretical reasoning, and obviously, this has to change. In other regions of the world, one could hardly study social reality without referring to organized violence. This holds for Mafia structures in Southern parts of Italy, for armed groups in Northern and Sub-Saharan Africa, for wars and dictatorships in the Middle East, for violent gangs and organized crime in Latin America, and for state violence in China and Russia.

In what follows we argue that violence always has been and probably will remain a crucial part of everyday life and of any type of social space. Violence is a crucial means of stabilizing and defending, but also of challenging and changing social order. In the twenty-first century, due to increasing numbers of authoritarian as well as fragile states, raising tensions for restructuring global power structures and swelling conflicts around scare resources and environmentally induced crisis, organized violence will be of growing relevance. Any sociological diagnosis of organized violence depends on an explicit and comprehensive conceptual framing of the term.

## The place of violence in sociology

Since the founding of sociology and social sciences in the ninteenth century, the topic of violence played a minor role in theory building and empirical studies. Violence was conceptualized either at the individual level as the expression of the intensity of social conflicts or at the state level as wars and “official” armed conflicts between sovereign entities.^8^ For critical sociologists, violence as the extensive use of armed forces reflected the rationally calculated interests of domination typical for colonial and imperial conquest, empires, and states. In modern capitalist societies, violence would be canalized in sophisticated channels of economic control and domination, and organized violence as wars would be the exceptional outbreak for rearranging power relations by state actors.^9^ For modernization theorists, open violence was supposed to be increasingly encapsulated and democratized in formalized structures and procedures of interest regulation. International law and institutions as created after World War I would, gradually, replace violent mechanisms of conflict resolution by formal and legal procedures. Legal state authorities would increasingly monopolize organized violence.^10^ In both (critical and modernization) approaches sketched out so far, organized violence is reduced to specific exclusive areas of societies. In an opposite extreme, authors like Johann Galtung extended or even diffused the concept of violence into all social relations when he defines “violence as avoidable insults to basic human needs, and more generally to life, lowering the real level of needs satisfaction below what is potentially possible” (Galtung, 1990, p. 292). By this, Galtung “extends the concept of violence so that any social system of social inequality that leads to unnecessary death is considered violent, even if this does not involve the deployment of physical force” (Walby, 2012, pp. 101f).

Independently of the specific (critical, modernist, or diffuse) approach, for decades the topic of violence only appeared marginally in social sciences (Walby, 2012; for political science: Barnes, 2017). In many dictionaries, the term violence was not treated explicitly.^11^ Although stating general ambiguities and tendencies between violence and non-violence, Flechtheim (1969, p. 374) refers to democratic states of law, constitutions, and culture, and to the claims of eliminating violence out of families and schools, and cites a “law of decreasing violence.” In their “Critical Dictionary of Sociology” Boudon and Bourricaud (1992, p. 182) argue: “In the long term there could be observed successful pacification processes between states as well as inside states.” Kaldor (2004, p. 153) states that “The nation-state had bottled violence—removed violence from domestic relations and bottled it up to be released in intense blasts known as war. Fear and superstition were channeled into external threats and enemy stereotypes”.

In general, focusing only on the assumptions of ubiquity, disappearance, or social context relatedness of violence, we can distinguish three different approaches on violence in sociology.^12^ One is to simply marginalize violence as a theoretically and empirically irrelevant topic. If violence is a pathological individual behavior like a disease, individual and collective violence should be treated as an abnormality and be handled mainly by physicians, psychologists, or criminologists, and only exceptionally by sociologists. “Humans are hard-wired to get caught in a mutual focus of intersubjective attention, and to resonate emotions from one body to another in common rhythms. This is an evolved biological propensity (…) to create interaction rituals and thus to keep up face-to-face solidarity. (…) We have evolved to be hyper-attuned to each other emotionally” (Collins, 2008, pp. 26f). If all individuals and their living together is predetermined for solidarity and peaceful interaction, violence could be considered an interesting, but marginal occurrence in social practice. In the perspective of modernization theory and individualist interaction, violence will be increasingly controlled by states and public authorities. “The marginalization of violence within core debates in postwar sociology is linked to development of the thesis that violence declines with modernity” (Walby, 2012, p. 97; for this debate see also Malesevic, 2014, pp. 66ff).

A second approach starts from the opposite extreme and characterizes violence as an evolutionist constant of “the human nature,” as a ubiquitous means to pursue individual or collective goals and as a relentless phenomenon in the history of mankind. In this perspective, violence is not the opposite of culture, but an inextricable part of it (e.g., Sofsky, 2003; von Bredow, 2018). Most of the partisans of this approach depart from Hobbes' assumption of mankind as characterized by *homo homini lupus est* (the man is the other man's wolf). In this perspective, violence is an endemic part of human nature. It could be controlled and fenced only by social orders as hierarchies and delegation of control over violence. “Our point of departure is a world of endemic violence. The anthropological literature on primitive societies suggest that most primitive societies were extremely violent. Most modern societies experience episodic civil war and breakdown of order, and the twentieth century proved one of the bloodiest of all times” (North et al., 2006, p. 10). The authors link the control of violence to the mechanism of limiting (economic) gains for the powerful groups in exchange for generalized restrictions of using violence for all, guaranteed by public or state authorities: “the formation of the state provides a first order solution to the problem of limiting violence by inducing the most powerful members of society to create arrangements that reduce their potential gains from using violence” (North et al., 2006, p. 12). In a modernist and pro US-American perspective, they then conclude that successful economic development is dependent on developing institutional arrangements that control violence and regulate access to resources either as limited access order (“by political manipulation of the economic system to generate rents by limiting entry”) or as open access order (“through political and economic competition rather than rent-creation,” North et al., 2006, pp. 4f).

In a third perspective, we could argue that violence is neither a disappearing or irrelevant issue nor an always present basic human drive or constant. Violence is an expression and part of human action and social practice developed in social entanglements. It is neither “naturally” omnipresent nor “naturally” vanishing, but its forms and dynamics depend on the social spaces in which human beings develop their interrelations with nature, others, and themselves. “Historical comparisons show that social organization is a huge component in determining the amount of violence that takes place” (Collins, 2008, p. 28). Evolutionary anthropological research suggests that the extent and forms of violence changed in a U-shaped trajectory: from high levels of violence at the transition from primates to *homo sapiens* toward lower levels in nomadic and hunter-gathering societies to gain increased levels in complex residential, food storing, agricultural, and densely populated societies (Knauft et al., 1991).

Concentrating on concepts of violence in modern societies, a starting point could be Max Weber's definition of a state as a “human community that (successfully) claims the monopoly of the legitimate use of physical force within a given territory.” (Weber, 1919, p. 4). Here, legitimate force or violence (Gewaltsamkeit) is considered as exclusively concentrated by the state. This follows Hobbes' idea of the state as an outcome of citizens' free decision to delegate certain rights and aspects of individual sovereignty to the Leviathan in order to guarantee the absence of physical violence in human relations. From that perspective, in civilized “human communities,” violence, independently of its origin and cause, should be eliminated out of direct social relations and monopolized by the public authority. For Weber, the distinction between power and dominance is crucial. Whereas power is defined as the chance or opportunity that someone can force his own (unspecific) will and orders against someone else even against his or her own will, domination means the chance or opportunity that someone obeys to the (specific or unspecific) orders of another in a way that, to a certain extent, the order appears as the own will of the subordinated. For Weber, domination always has an element of disposition to obey (Gehorchenwollen). This claim of legitimacy could be based on rational calculations, on charismatic or emotional guidance, or on established traditions and routines.

For Weber, a defining criteria of sovereign states is their claim and capacity to exercise the legitimate monopoly of control over physical violence within a given territory. This validity of legitimacy (Legitimitätsgeltung) could be based on legal-rational, charismatic, or traditional domination. According to Weber, in the case of pure power execution, there is no need for such a legitimacy but the chance to impose one's own will, even against the will of another person, is based on violence and force. Violence can be considered an important means to implement power against the will of the thereby subordinated; meanwhile, domination requires legitimate use of violence by state sovereigns. In this classic perspective, physical violence is the practice of physical harm against human bodies and their valuables by using physical body force or artifacts like weapons. Modern society claims to be a social order where physical violence in its very different forms is absent and monopolized by the state. Violence, except the legal violence of states, is considered something abnormal, exceptional, to be eliminated. This is why in sociology violence is marginalized as a topic of theory and research. It is normally treated in the context of “deviant behavior,” but not embedded in general concepts of social praxis, everyday life, organizations, and institutions.^13^

Overcoming such classic focus on formally organized violence of states and their military, more recently several scholars state a new quality of warfare. Münkler (2005, p. 3) ascribes three characteristics to what he coined “The New Wars”: denationalization, greater asymmetry of military force, and increasingly autonomous forms of violence. In her seminal book “New and Old wars,” Kaldor (2012) underlines that the traditional understanding of war as applying (legitimate and legal) violence between sovereign states must be revised and extended. The classic definition of states as having the legitimate monopoly of violence is no longer sufficient and has been eroded “from below” (Kaldor, 2012, p. 6) as non-state military groups claim legitimate use of organized violence. According to Kaldor, new wars are more about identity politics (with aims justified by ethnic, religious, or cultural arguments) than about geo-strategic or ideological reasons. Concerning the new methods of warfare, Kaldor holds that classic wars focused on conquering or defending territory by military means; new wars apply a mixture of guerrilla tactics and counter-insurgency methods (Kaldor, 2012, pp. 9f, 71ff). Finally, new wars are characterized by decentralized, informal, and global mechanisms of financing. Whereas classic wars had central and autarchic financing controlled by nation-states, new wars use “plunder, hostage-taking and the black market” (Kaldor, 2012, pp. 10 and 94ff).

For the field of political science—and this holds also for sociology—Barnes argues that in the study of organized violence state violence, political violence, and criminal violence have to be analyzed integrally: “continuing exclusion of organized criminal violence from the literature on political violence is neither empirically nor theoretically justified” (Barnes, 2017, p. 968). For deeper insights into the relations between states and violent organizations, he proposes four ideal typical constellations: confrontation, enforcement-evasion, alliance, and integration (Barnes, 2017, pp. 973ff). Malesevic (2014) already argued that the tendencies of declining, ongoing, or increasing organized violence only could be analyzed from a long-term perspective. According to this, the eighteenth and nineteenth century witnessed a decline in warfare, but “the current decline of warfare is in fact a temporary phenomenon rooted in the same organizational logic that shaped our world over the last twelve millennia. In other words, instead of reflecting a profound and permanent shift in historical development and a significant change in human attitude to war the contemporary decrease of organized violence is a product of specific geo-political and organizational constellations” (Malesevic, 2014, p. 83).

In sum, answers to the question if organized violence is decreasing, increasing, or simply shifting its forms depends on the more specific understanding of the concept of violence in general. The broadest approach was probably proposed by Johan Galtung. He defines violence as “present when human beings are being influenced so that their actual somatic and mental realizations are below their potential realizations.” (Galtung, 1969, p. 168). This is such a broad definition that it includes almost all social relations. Nevertheless, the typology of violence proposed by Galtung (1969, pp. 169–173) could be used to define more specific concepts. He differentiates (1) physical from psychological violence, (2) negative from positive means of influencing, (3) violence with or without objects that directly hurt, (4) personal from structural violence, (5) intended from unintended violence, and (6) manifest and latent violence.^14^

On the other extreme, there are efforts to address violence only in a quite specific and war-related manner. In a political science perspective, Finlay (2018, pp. 361f) defines violence as “the intentional infliction of (severe) harm by human agents on others usually effecting itself in physical injury in paradigm cases but (on some accounts) also encompassing psychological damage. Acts of violence are typically also descriptively violent in that they are sudden, forceful, and sensational. Harms are inflicted by directing such actions toward either a victim's body or something they value (such as their property).” He elaborates that violence always has three features. First, it includes agency and actors that aim at harming in an intentional action. Second, it relates to bodily or physical harm; this could lead to other kinds of harm like psychological injury, but the starting point in this understanding is physical harm. Third, violence is and should be observable in a descriptive sense as an act, as a (sudden, forceful, and tangible) event. Finlay discusses the possibility to include cyber-attacks in such a specific understanding of violence and concludes that cyber-attacks could even be understood as elements of Just Wars.^15^

In social sciences, concepts of violence normally are located between such extremes of very broad or quite specific understandings. In a sociological perspective and in line with Popitz (2017), we could define violence as the *executed or convincingly threatened social action of intended harming physically and/or injuring mentally of oneself, another person, or a group of persons*.^16^ Such a definition is broad enough to include physical and mental injuries because in the twenty-first century, in light of the central role of social media use and corresponding aggressive harassment, it would be inadequate to only include direct physical force (that even is not in place in the cited case of cyber-force). At the same time, it is specific enough in the sense of focusing on *social action of (successfully or unsuccessfully) intended harming that could be actually set into practice or could be announced in a credible way and thereby influence social action*. Especially, in the context of organized violence and *violent social spaces*, which will be dealt with in the following section, it is crucial to consider not only the actual execution of physical harm but also the convincing threat with it, so that it has social consequences (see e.g., Keller et al., 2017, p. 5). Based on this, we understand *organized violence* as *putting into practice or convincingly threatening with social action that harms persons or groups physically and/or injures mentally in a collective way in order to achieve collective and/or corporate goals*.^17^ Organized violence encompasses violence perpetrated by constituencies like nation-states as well as collective or corporate actors, legal and illegal, with varying levels of legitimacy. While the concept of *organized crime* focuses on the aspects of legality/illegality and on business-like organizational structures, we use the term *organized violence* for focusing on a broader variety of goals (including religious or ideological ones) in relatively stable arrangements of the division of labor, hierarchy, and membership. The concept of organized violence differs from the general term violence by stressing the organizational aspects. Organizations are more or less durable, vertically and horizontally differentiated entanglements of cooperating members.^18^

There are many proposals for differentiating forms, motives, causes, actors, and aims of violence.^19^ Only a few can be mentioned here that help to substantiate the concept of organized violence. As a general phenomenology of physical violence, Reemtsma (2012) differentiates the three types of autotelic violence (that aims at *destroying or extinguishing* bodies and persons, e.g., killing, wounding etc.), raptive violence (that aims at *using and exploiting* bodies and persons, e.g., sexual violence, forced labor etc.), and (dis)placing violence (that aims at shifting the location and home of bodies and person, e.g., either by *capturing/enclosing or by displacement, expulsion*, etc.). In his sociological analysis of violence, Collins (2008) underlines the micro-dynamics of violence as complex social interactions that develop a momentum by their own, although they are embedded in broader social structures like social classes, experiences of racist and gender discrimination or of desperation. Collins focuses on violence as generated in and generating situations of co-presence and action dynamics.^20^

Such micro-analysis could help to differentiate and extend the predominantly treated rationalist aspects on the use of violence. Since the latter could be applied for the control of spatial resources like territories of social resources, such as legitimacy and reputation, of economic resources like money or value chains, of political resources like influence and power, situational violence also has its own momentum and dynamics. Violence can be situated in rational strategies (like criminal or war violence) or in institutional arrangements of inequality and discrimination (like gender or ethnic violence). But it also can gain its own momentum as collective violence that is out of control (like in riots and side effects in almost all wars).^21^ Based on the protocols of everyday communications of imprisoned German soldiers of the German Nazi regime in the United Kingdom, violence has been analyzed in its combination of bureaucratized and industrialized genocide plus barbarism and eruptive human slaughter (Neitzel and Welzer, 2011). Until deeper research, Russian troops' behavior in Ukraine, e.g., in Bucha in April 2022, seems to show up elements of this mix of organized rational cool calculus and spontaneous unleashed cruelty.

## Organized violence in social spaces of everyday life

As demonstrated in the preceding section, in a sociological perspective, violence, especially organized violence, needs to be analyzed not as abstract human traits or “natural” characteristics but as embedded and (re)produced in social relations and social orders. Interstate wars and most other types of state-based conflicts (see Figure 1) aim at redefining or defending power relations, borders, and governments, in sum: a specific social order of and between states. Organized violence that originates in such contexts is easy to identify, and people fleeing from it normally are—at least in the first moment and due to the visibility of violence—well received in neighboring and other countries. This was the case in the Yugoslavian War in the 1990s, during the Iraq War in the 2003, the civil war in Syria in 2011, and more recently during the war in Ukraine in 2022. A general pattern is that people who flee such an open armed conflict think and plan to return in a reasonable period, like some months. Also, the receiving states consider their assistance to refugees as a transitory emergency measure.^22^ In all these cases, armed conflicts and organized violence are considered and treated as exceptional and transient phenomena in social spaces that normally are *not violence-intensive*.

Things get much more complicated, when *violence-intensive social spaces* (ViSS) of warfare, armed conflicts, and organized violence lengthen in time so that people step by step arrange this situation as a taken-for-granted social order.^23^ Then return migration gets more complicated or impossible, and conflicts in places, where forced migrants arrived and rested temporally, often increase due to the lack of resources (Erdogan, 2019; Rottmann and Kaya, 2020). Forced migrants are then stuck in the limbo of not being able to return, of not being able, and/or willing to stay definitely in their current places and of not being able to move onward to other places. Such complex situations of protracted organized violence and forced migration will be of increasing relevance in the future.^24^ Ríos (2014, p. 200) underlined that “we need to broaden our analysis of the factors that we normally analyze as part of traditional immigration […] academics should bring attention to how security environments affect relocation.” Natural disasters and the impacts of climate change often originate organized violence as armed conflicts, ethnically grounded violent confrontations or organized crime plays. This fuels forced migration and protracted displacement that in turn could lead to enhanced organized violence.

In this context, the distinction between *violence-intensive social spaces* (ViSS) and *not violence-intensive social spaces* (NoViSS) seems helpful for grasping the corresponding challenges. Whereas the term “spaces of violence” was used in Kalyvas (2006) and Baberowski (2015) in a geographical or unspecific way, Koloma Beck (2017) applies the concept of space in a more explicit manner of everyday life.^25^ Based on the concept of lifeworld and her ethnographic fieldwork in Kabul/Afghanistan, she analyzed the situation of war as the subjective experience of taken for granted everyday social practices. For social actors, violence and security issues generate topological orders, as places, borders, walls, and zones that create their own logics of action. Although we could characterize the city of Kabul as a ViSS, people living there develop their own “perceptive, cognitive, emotional and action practical routines of managing” (Koloma Beck, 2017, p. 21) the city. Their cognitive maps distinguish places and social settings as either secure or dangerous. By this, they develop “archipelagoes” of their life world and make safe places by physical artifacts and “bunkerasition” (like walls, secure houses), by social rules and practices (of controlling and restricting access to places) and using systems of symbols (like dress codes, languages, food). The study of Koloma Beck demonstrates that violence and security are integral elements of the social spaces of everyday life.

NoViSS and ViSS should be considered as opposed to ideal types on a continuum of social spaces, where violence always is in play, but in substantially different constellations of actors, means, legality, and legitimacy. Since the second half of the twentieth century, most early industrialized countries in Europe and North America perceived themselves as having successfully developed general environments of state monopolies of legal and legitimate violence, even if outreaching state violence against marginalized groups or organized violence of gangs and criminal networks like mafias still were strong. Except during World War I and II, most people seemed to live their everyday life in NoViSS. Obviously, each society and locale has specific dark places, e.g., beyond central railways stations that at night by many are perceived as exceptionally violent and unpredictable social spaces, but in turn, might be seen as taken for granted and manageable social spaces by those living or working there.

Even during the German Nazi regime until the beginning of World War II, a great portion of the society might have experienced their own life as occurring in NoViSS, while Jews, homosexuals, unionists, and leftist activists suffered in ViSS. The industrialized terror and genocide against specific ethnic, religious, political, and gender groups was staged as legalized and was experienced by most supporters and sympathizers of the regime as legal *and* legitimate.^26^ During its first years, the Nazi regime in Germany could be characterized by the coexistence of NoViSS for many people and ViSS for specific groups. The relation then developed to the “total war” as ViSS for all. Nowadays, some people living in gated communities with extended security services might feel to live in NoViSS, whereas other social groups living in the same city will experience it as ViSS.^27^ This underlines that organized violence is not only an objective matter expressed in the number of armed conflicts or fatalities but also a subjective perception and everyday awareness of vulnerability, stress, and threats. In sociology, we lack internationally recognized instruments to measure both, the objective and the subjective side of organized violence. Even for the objective aspect, there are only a few time series of data that are comparable, and these longitudinal data sets only present indicators at the level of states, whereas ViSS and NoViSS could differentiate at the local or regional level. Therefore, the two indicators dealt with in the following, the number of intentional homicides and of armed conflicts, represent only a first approximation.

**Figure 2:**
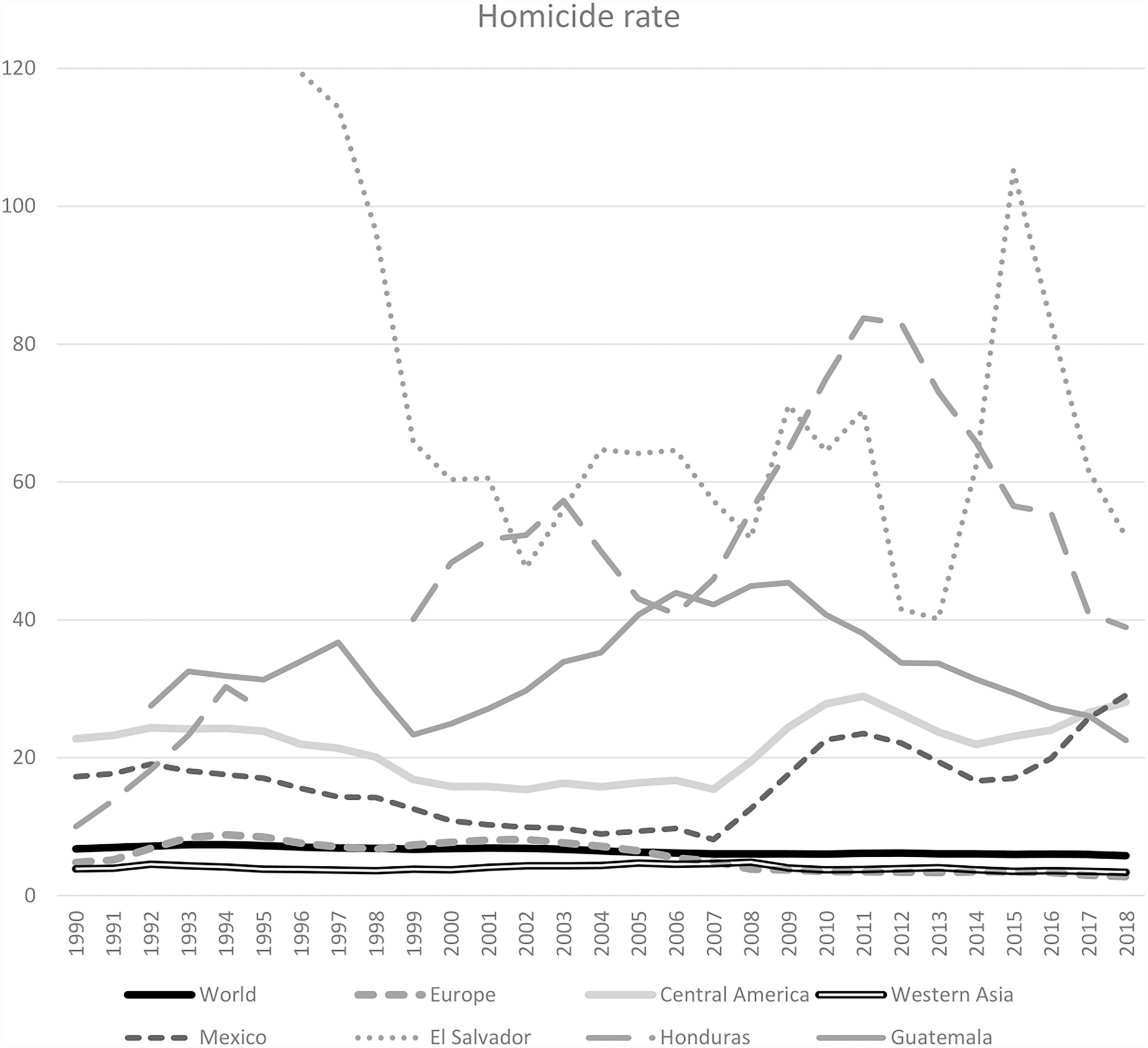
Homicide rate: world, regions, and countries. Source: Own elaboration based on https://dataunodc.un.org/data/homicide/Homicide%20victims%20worldwide.

As a first proxy for ViSS, we could take the share of homicides per 100,000 inhabitants. There are related data over longer time periods. Besides the UCDP project, the United Nations Office on Drugs and Crime (UNODC) collects data about intentional homicide rates by countries.^28^ These are based on reports of the member states themselves and therefore reliable to the extent of the credibility of national information. By this, we have comparative data at least for some countries and regions on an annual basis since 1990.^29^ In a first approach and based on UNODC data for regions we can identify substantially different levels of estimated rates of intentional homicide for El Salvador, Honduras, Guatemala, Mexico as compared to the World, to Europe, and to Western Asia.^30^ Figure 2 reflects the continuity of ViSS in Central American countries and Mexico and almost NoViSS in Europe and Western Asia.^31^ During this period, the estimated worldwide homicide rate oscillated between six and seven fatalities per 100,000 inhabitants, in Western Asia (counting 18 countries from Armenia up to Yemen, including Syria and Turkey) it was about four, in Europe it decreased from 4.8 to 2.8. But in Mexico, it started in 17.3 in 1990 and grew up to 29 in 2018. During all three decades, the level of homicide rates in Central America has been more than four times higher than in all other regions under consideration, and after a decreasing tendency it almost doubled since 2008 and until 2018. In 2018 Mexico took over the historically ever high level of Central America. But extremely high are the homicide levels for Guatemala, Honduras, and El Salvador. In El Salvador, it reached 142 in 1995 (cut off in the scale in Figure 2).

**Figure 3:**
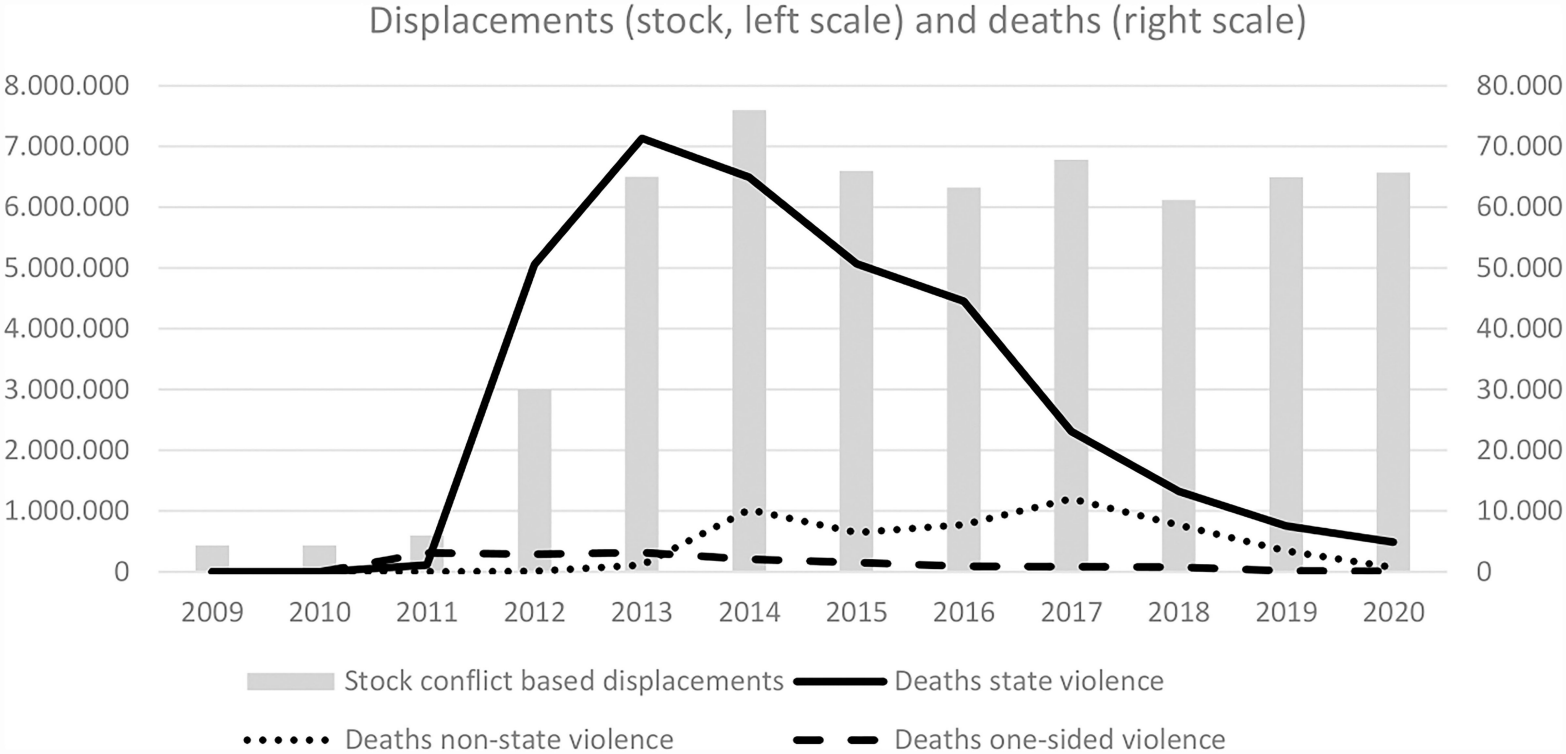
Conflict-based displacements and deaths in Syria 2009–2020. Source: Own elaboration based on https://ucdp.uu.se/country/652 and https://www.internal-displacement.org/database/displacement-data

Much of this violence has to be considered organized violence (Cruz, 2011; Bowen, 2019). According to these data, since the 1990s and independently of turbulent violent contexts (e.g., in Lebanon, Israel/Palestine, Iraq, etc.), Western Asia could be considered as NoViSS as compared to Central America or Mexico (Figure 2). After 2010, this situation changed dramatically in Syria, when the regime began to suppress protest movements and emerging armed groups with organized violence. Massive emigration began, mainly to Turkey and afterwards to the European Union. In social sciences there is broad consent to characterize this as forced or refugee emigration from ViSS. The intentional homicides officially reported by the Syrian government to the UNODC are listed only for the years 1997 to 2010 and oscillates between 4.5 and 5.5 homicides per 100,000 inhabitants.^32^ Here, the data of the UCDP can offer a more detailed picture of the development of deaths due to organized violence executed by the State, by Non-State, and by One-Sided actors. Figure 3 differentiates the UCDP estimates according to the three sources of deaths and reveals the prevalence of the Syrian state as the main cause of organized violence since 2012. From 2012 to 2018, Syria had extraordinarily high levels of deaths due to state violence. Since then it has ranges (again) below the level that Guatemala, El Salvador, and Honduras represent for three decades.

The two examples of indicators of violence in social spaces underline the usefulness to differentiate between NoViSS and ViSS. They also show that there could be a high level of ViSS during a period of several decades, like in Central America, but that there also could occur a disruptive change from NoViSS to ViSS like in the case of Syria. These estimated data of (organized) violence are crucial as objective criteria, but especially their changes over time may substantially influence subjective perceptions of actors. They influence what is realized as “normal” and as part of everyday life “until further notice.” Here, organized violence is present by actually exerting power, but also by *convincingly threatening social action of intended harming physically or injuring mentally*. Therefore, social spaces are perceived and socially constructed as ViSS or as NoViSS at the level of social actors in their everyday practice. Consequently, the concept of *mixed migration* refers not only to varying objective reasons (like “voluntary economic” and “violently forced”) but also to composed subjective significance. Such social perceptions and meanings of the degree and role of violence in everyday life can vary substantially, as can be seen comparing Syria and Central America.

In the Middle East, armed conflicts between states, military interventions, and violent dictatorship have occurred for decades. But for Central America, there also exists ViSS based on military coups, dictatorship, armed guerilla conflicts, organized crime, drug cartels, and violent youth gangs since generations. Taking the Middle East and Central America as examples, the nature, spread, and durability of organized violence show similarities and variations, but in both cases, countries as a whole and specific regions therein pulsated between NoViSS and ViSS. When in a short period of time, organized violence increases substantially (like in Iraq in 2003 or in Syria from 2012 and 2018), for most people this shifts the role of violence from NoViSS as a more or less predictable and calculable monopoly in a “normal dictatorship” toward ViSS as unpredictable multipolar organized violent groups defined by religious, ethnic, political, and/or economic agendas (e.g., Doyle and Dunning, 2018). In the Middle East, for a long period, strong states monopolized most organized violence, thereafter organized violence spread in “fragile states.” Nevertheless, compared to this, in Central America, organized violence is multipolar and multi-dimensional since mid of the twentieth century, that is, for many generations (Bowen, 2019).

## Concluding remarks

For a long time, sociology, especially European sociology, for various reasons did not draw much attention to the topic of organized violence. The Russian invasion of Ukraine and the atrocities against civilians force us to revise the role and our concepts of organized violence in the twenty-first century. Organized violence is not a relic of “backward societies” or marginal regions, but an endemic challenge in many places of the world. The manifold factors of increasing numbers of fragile states (Ziaja et al., 2019) and eroding democracy worldwide (BTI, 2022), of inter-state conflicts and military tensions, of transnationally organized crime, cartels, mafias and gangs, of enduring conflicts for resources due to climate change, and of increasing global social inequality lead to spreading and enduring of organized violence. This will be a releasing factor for internal or cross-border migration (like in the case of armed conflicts or ViSS). Ríos (2014, p. 210) “provided evidence supporting the idea that gaining a more complete understanding of migration outflows within Mexico and between Mexico and the United States requires one to account for the literature on organized crime violence.” Organized violence could be perceived as legitimate (like in regions controlled by armed groups or drug cartels) or illegitimate and/or experienced as an abrupt increase of ViSS. In both cases, it could trigger forced migration (e.g., Crawley and Skleparis, 2018). Effects of organized violence could be highly selective for specific groups (e.g., actors with high economic, cultural), and social capital could be more mobile (e.g., Castles, 2010). In sociology, we have to strengthen further empirical and theoretical research on these topics.

## Data availability statement

The original contributions presented in the study are included in the article/supplementary material, further inquiries can be directed to the corresponding author.

## Author contributions

All authors listed have made a substantial, direct, and intellectual contribution to the work and approved it for publication.

## Funding

Work for this article was supported by a grant from the German Research Foundation (Pr 637/14-1).

## Conflict of interest

The author declares that the research was conducted in the absence of any commercial or financial relationships that could be construed as a potential conflict of interest.

## Publisher's note

All claims expressed in this article are solely those of the authors and do not necessarily represent those of their affiliated organizations, or those of the publisher, the editors and the reviewers. Any product that may be evaluated in this article, or claim that may be made by its manufacturer, is not guaranteed or endorsed by the publisher.

## Footnotes

^1^I appreciate valid comments and suggestions of Rafael Bohlen and of the two reviewers.

^2^http://necrometrics.com/pre1700a.htm; see also White (2011) and Petterson and Eck (2018, pp. 537f); https://www.pcr.uu.se/research/ucdp/about-ucdp/. This paper was produced in the context of the research project “Organized violence, new migration patterns, and development: A comparative study in Europe and the Americas” that is funded by the German Research Foundation and runs from 2019 to 2022 (see https://www.migration-violence.org/); I appreciate the comments and suggestions of Melanie Wieschalla on a former version of this paper.

^3^For a first view see https://dataunodc.un.org/data/homicide/Homicide%20victims%20worldwide.

^4^See the charts on https://ucdp.uu.se/downloads/charts/.

^5^For an interesting concept and measuring of fragile states and the role of violence herein see Ziaja et al. (2019).

^6^Interestingly, in many introductory books on violence there is no explicit treatment of organized violence; collective violence is mentioned as criminal violence, organized crime or violence in armed conflicts, see e.g., Ray (2011), and Heitmeyer and Hagan (2002a).

^7^UNHCR (2011, p. 6, 2021, p. 10f); https://migrationdataportal.org/themes/international-migrant-stocks.

^8^For an extended handbook of violence, see Heitmeyer and Hagan (2002a). and therein the broad discussion of concepts, dimensions and definitions of violence of Imbusch (2002).

^9^As critique of this “classical understanding,” see e.g., Weiss and Six-Hohenbalken (2011, pp. 2f) and Shaw (2009, p. 105) embedded the concept of organized violence in Western or Northern discourses on war and violence: “The most fruitful macro-sociology of the last quarter-century has been the historical sociology of power and the state (…), with many implications for the study of violence, even if it often has dealt more with the role of violence (i.e. the power context) than the nature of violence (the character of war, genocide, etc.).” Shaw argues that a “historical theory of the transformation in the relationships of military power (violence) and other forms of social power” (Shaw, 2009) is needed. Based on this historical approach, he distinguishes new forms of war after the period of “industrialized total wars” as a “global surveillance mode of warfare, as the framework for armed violence of all kinds. This involves not only mutations in the forms of warfare—“new and old wars,” “new Western warfare,” “new global terrorism”—but also transformations in the relationships between organized violence and social power generally” (Shaw, 2009).

^10^Actually, not only Pinker (2011) argues for a modernization or civilization driven long-term decrease of violence; based on data estimates from the thirteenth up to the twentieth century, Eisner (2002, p. 63) demonstrates the going down of violence when measured as rates of homicides in different European regions.

^11^In his seminal essay, Reemtsma (2012, pp. 261f) terms it as “Sociology's Silence.” The Oxford Paperback Dictionary of Sociology of 1994 and 1998 did not include a definition of the word violence; the Cambridge Dictionary defines violence as “extremely forceful actions that are intended to hurt people or are likely to cause damage” (https://dictionary.cambridge.org/de/worterbuch/englisch/violence; the Merriam Webster Dictionary defines it as “the use of physical force so as to injure, abuse, damage, or destroy” (https://www.merriam-webster.com/dictionary/violence); a more differentiated definition is offered by the World Health Organization (WHO) as “the intentional use of physical force or power, threatened or actual, against oneself, another person, or against a group or community that either results in or has a high likelihood of resulting in injury, death, psychological harm, maldevelopment or deprivation.” [WHO (World Health Organization), 2014, p. 84].

^12^See for this distinction and other differentiations and typologies, e.g., Galtung (1969, 1990), Collins (2008), Wieviorka (2009), Malesevic (2010, 2014), and Walby (2012).

^13^For this argument, see Sofsky (2003) and Reemtsma (2012).

^14^His concepts of cultural and structural violence (e.g., Galtung, 1990) make it almost impossible to separate violence from any kind of social practice.

^15^For the general distinction of just and unjust wars, see Walzer (2015).

^16^See also Heitmeyer and Hagan (2002b, p. 23), who insist that violence is not just a mean but an outcome of social processes.

^17^Interestingly, in the ample “International Handbook of Violence Research” (Heitmeyer and Hagan, 2002a) the concept of organized violence is of minor importance, the more than 1,500 pages book has no specific chapter on this topic. In a “Guide for the Red Cross and Red Crescent” organized violence is defined as “the purposeful and systematic use of terror and brutality to control individuals, groups and communities […] Its methods include causing severe pains and suffering, killing, intimidating, threatening and in some cases destroying a community, thence group or political opposition. Government usually direct military, police and political organizations to perform organized violence, or those groups may act indecently in the persecution of specific individuals, groups and communities” (Kane, 1995, p. 5). This is a valuable definition, but it is too narrow for the context of forced migration, where border control agencies, human traffickers or professional smugglers might be active without high levels of brutality and terror.

^18^The concept of organized violence is also used by the Uppsala Conflict Data Program for defining incidents of organized violence as their units of analysis: “The incidence of the use of armed force by an organized actor against another organized actor, or against civilians, resulting in at least 1 direct death in either the best, low or high estimate categories at a specific location and for a specific temporal duration.” (Sundberg and Melander, 2013, p. 524).

^19^See e.g., Heitmeyer and Hagan (2002a)., Ray (2011); for good recent overviews only in Germany, e.g., von Trotha (1997); Sofsky (2003); Gudehus and Christ (2013); Baberowski (2015), and Hoebel and Knöbl (2019).

^20^In a similar perspective and against exclusively rationalist explanations of the use of violence (Fujii, 2013, p. 413) analyzed the massacre of My Lai in Vietnam, the Rwandan genocide and y lynching in rural Maryland/USA in order to explain “extra-lethal violence” using a performative analysis approach. “Performative analysis adds to this array by embedding strategic calculation, meaning-making, and social dynamics within a particular kind of social process—the process of putting violence on display for gruesome effect.” She explains e.g. the violence of forcing people to dance before being killed as part of a processual, endogenous and autonomous spiral of violence.

^21^Similarly, the concept of ethnic violence proposed by Malkki (1995); Appadurai (1998) analyzed the ethnically constructed violence between Hutu and Tutsi in Rwanda and Burundi.

^22^A recent example is the forced migration from Syria to Turkey (see e.g., Erdogan, 2019; Içduygu and Nimer, 2019; Rottmann and Kaya, 2020).

^23^Baczko and Dorronsoro (2022) apply Bourdieu's concepts of state, capital and habitus to situations of (protracted) civil wars and corresponding conflicts between and shifting of social orders. For the concept of social spaces (see Pries, 2001, 2005).

^24^See e.g., Turton (2003, p. 8) and (International Migration Organization (IOM), 2019).

^25^For the concept of everyday life worlds, see Schütz and Luckmann (1973); also the volume edited by Six-Hohenbalken and Weiss (2011).

^26^See e.g., Bialas (2012) and Reemtsma (2012).

^27^For a similar argument based on Bourdieu's theory of social practice (see Baczko and Dorronsoro, 2022, p. 211).

^28^For methodic problems of statistical data on crime and violence (see e.g., Ray, 2011, pp. 64ff).

^29^Alternative data could derive from UCDP or by the IDMC-GRID (Internal Displacement Monitoring Centre) (2020); but both data sets do not include longer time series and have a narrower approach; see https://www.internal-displacement.org/about-us; for UCDP see https://ucdp.uu.se.

^30^See https://en.wikipedia.org/wiki/United_Nations_geoscheme_for_Asia#Western_Asia. For the general migration context of Middle-Northern America (see Pries, 2019).

^31^Obviously, as mentioned before, there might be gated communities in El Salvador with less (organized) violence than in specific neighborhoods of the USA, France or Germany; but the general level of differences between countries over longer time periods is salient.

^32^See https://dataunodc.un.org/data/homicide/Homicide%20victims%20worldwide.
